# Ten-Year Analysis of Bacterial Colonisation and Outcomes of Major Burn Patients with a Focus on *Pseudomonas aeruginosa*

**DOI:** 10.3390/microorganisms12010042

**Published:** 2023-12-25

**Authors:** Jenny Gomersall, Kalani Mortimer, Deniz Hassan, Kathryn A. Whitehead, Anthony J. Slate, Steven F. Ryder, Lucy E. Chambers, Mohamed El Mohtadi, Kayvan Shokrollahi

**Affiliations:** 1 Whiston Hospital, Mersey and West Lancashire Teaching Hospitals NHS Trust, Prescot L35 5DR, UK; 2 Department of Microbiology, Whiston Hospital, Mersey and West Lancashire Teaching Hospitals NHS Trust, Prescot L35 5DR, UK; 3 Mersey Burns Centre, Whiston Hospital, Mersey and West Lancashire Teaching Hospitals NHS Trust, Prescot L35 5DR, UK; 4 Microbiology at Interfaces, Manchester Metropolitan University, Chester Street, Manchester M1 5GD, UK; 5 Department of Life Sciences, University of Bath, Bath BA2 7AY, UK; ajs319@bath.ac.uk; 6 Department of Biology, Edge Hill University, Ormskirk L39 4QP, UK; 7 Manchester Metropolitan University, Chester Street, Manchester M1 5GD, UK; 8 University of Liverpool, Foundation Building, Brownlow Hill, Liverpool L69 3BX, UK

**Keywords:** *Pseudomonas aeruginosa*, burns, mortality, environment, infection, polymicrobial

## Abstract

A retrospective descriptive study included patients admitted with severe burns over the course of 10 years (2008–2018). Across all patients, there were 39 different species of bacteria, with 23 species being Gram-negative and 16 being Gram-positive bacteria, with also five different species of fungi cultured. *Pseudomonas aeruginosa* was the most commonly isolated organism, with 57.45% of patients having a positive culture. There was a significant difference in the number of *P. aeruginosa* isolated from patients that acquired their burns at work, in a garden, inside a vehicle, in a garage or in a public place. In patients that were positive for *P. aeruginosa*, the number of operations was higher (2.4) and the length of stay was significantly increased (80.1 days). Patients that suffered from substance abuse demonstrated significantly higher numbers of isolated *P. aeruginosa* (14.8%). Patients that suffered from both mental health illness and substance abuse demonstrated significantly higher numbers of *P. aeruginosa* isolated (18.5%). In the *P. aeruginosa*-negative group, there were significantly fewer patients that had been involved in a clothing fire. Furthermore, in the *P. aeruginosa*-negative patient cohort, the mortality rate was significantly higher (*p* = 0.002). Since the incidence of *P. aeruginosa* was also associated with a decreased mortality rate, it may be that patients admitted to hospital for shorter periods of time were less likely to be colonised with *P. aeruginosa*. This study demonstrates novel factors that may increase the incidence of *P. aeruginosa* isolated from burn patients.

## 1. Introduction

Thermal injuries such as burns represent a major clinical issue that causes an enormous burden to healthcare services, demanding increased medical efforts and a substantial amount of healthcare funds. Burns are a serious global public health problem with the World Health Organisation (WHO) estimating 11 million people per annum worldwide suffer from a burn injury. In the UK, an average of 13,000 people each year are admitted to hospital with a burn injury and an estimated 400,000 are injured from burns in the USA leading to 40,000 admissions to hospital. The resource demands that are related to this volume and severity of injuries is substantial [1]. Burns often become colonised by bacteria due to the loss of the cutaneous barrier, the presence of denatured proteins and lipids and immunosuppression [2]. This leads to substantial mortality rates with reports indicating that 42% to 65% of deaths in patients with burns are attributable to infection [3].

Burns are frequently colonised with a diverse range of Gram-positive and Gram-negative bacteria such as *Staphylococcus aureus*, coagulase-negative *Staphylococci* spp., *Pseudomonas aeruginosa*, *Enterococcus* spp., Vancomycin resistant *Enterococcus* spp., *Escherichia coli*, *Klebsiella pneumoniae* and *Acinetobacter* spp. [2]. *P. aeruginosa* is commonly detected at later stages in chronic wounds, with a predominance in burn wounds [4]. *P. aeruginosa* is resistant to a wide range of antibiotics and has the ability to acquire additional resistance after failed treatments regimes; resistance has been observed to many common first-line antibiotics, including β-lactams, polymyxins, aminoglycosides and quinolones [5]. In addition, *P. aeruginosa* has the ability to produce biofilms in several niche environments including burns [6]. Biofilms have a specific complex architecture and structure with biochemical characteristics that result in an increase in resistance against host immune mechanisms and antibiotics [7]. The presence of bacteria in cocultures was also demonstrated to increase their synergistic interactions affecting factors such as virulence, colonisation and persistence traits [8]. For example, it was demonstrated that when *P. aeruginosa* and *S. aureus* were grown in coculture, their antibiotic tolerance was enhanced [8]. Coinfection between the priority burn-wound pathogens is common, with *P. aeruginosa* and *S. aureus* frequently being coisolated [9]. Therefore, it is imperative to characterise the different bacterial species that can colonise burn wounds in order to further elucidate any synergistic effects.

Several risk factors that contribute to the development of bacterial infections in patients with burns have been documented. These include the location of burns and the age, gender and health conditions of patients with burn trauma. The present study aimed to determine the possible common risk factors in severe-burn patients that became colonised with *P. aeruginosa*, using retrospective data over a 10-year period (2008–2018). Findings from this work will lead to a better understanding of the underlying factors that cause *P. aeruginosa* to be isolated from patients with burn wounds.

## 2. Methods

### 2.1. Patient Eligibility

The present study represents a retrospective descriptive study on patients with severe burns admitted to the burns unit of a National Health Service (NHS) hospital situated in the north of England from 1 January 2008 to 31 August 2018. This unit serves 4.5 million people and has 12 beds plus intensive care unit (ICU) support. Patients were eligible for inclusion if they were admitted with >40% burns during the time period and had accessible clinical records and a date of discharge. The International Burns Injury Database (iBID) was used to identify patients coded as admitted with severe burns, and patients with toxic epidermal necrolysis (TEN) were excluded.

### 2.2. Defining Common Factors

A list of potential common factors was created through an initial review of patient records, a literature review and clinical reasoning. Telepath, EMIS and iBID were used to access patient records and collect data.

### 2.3. Statistical Analysis

A statistical package (SPSS version 27) was used for all data analyses. Patients were divided into two statistical groups which included patients with at least one positive *P. aeruginosa* culture and patients without a positive *P. aeruginosa* culture. To assess age and total body surface area (TBSA) against mortality in positive and negative patients, these groups were subdivided to create four groups, *P. aeruginosa*-positive patients that survived, *P. aeruginosa*-positive patients that died, *P. aeruginosa*-negative patients that survived and *P. aeruginosa*-negative patients that died. Where applicable, Pearson’s chi-squared with phi coefficient was used for categorical data, Mann–Whitney U test and independent t-test were used for nonparametric and parametric continuous data, respectively, and Kruskal–Wallis test and One-way ANOVA were used for comparisons between more than two groups using nonparametric and parametric data, respectively. Odds ratio, ANOVA and t-tests were also used. All tests were carried out at the 5% significance level with *p* < 0.05 indicating significant differences between groups.

## 3. Results

During the ten-year period, 53 patients were coded as being admitted with severe burns. Out of these, 47 were eligible for inclusion in this study. Patients were split into two groups, those with a positive *P. aeruginosa* culture (*n* = 27, 57.5%) and those without a positive *P. aeruginosa* culture (*n* = 20, 42.5%). The mean age of all patients was 47.5 years (SD ± 18.2), and this was not significantly different between groups (U = 237.00, *p* = 0.48). Most patients in both groups were male (66.0%), and no significant difference was observed between groups (Χ^2^(1) = 0.25, *p* = 0.62).

### 3.1. Factors Tested

Factors that demonstrated significant differences included the species of microorganisms determined, the environment at the time of injury, if the burn involved a clothing fire, number of operations, the length of hospital stay and if the patient suffered from mental health illness and substance abuse (Table 1). It should be noted that in some cases, the numbers were too small to infer significance.

### 3.2. Species of Microorganisms

Across all patients, there were 39 different species of bacteria, with 23 species being Gram-negative and 16 being Gram-positive bacteria, with five different species of fungi also cultured (Table 2). *P. aeruginosa* was the most commonly isolated microorganism, with 57.45% of patients having a positive culture. Other commonly cultured microorganisms included *S. aureus* (46.80%), *Enterococcus faecalis* (36.17%), *Escherichia coli* (31.92%), *Candida albicans* (27.70%) and *Enterobacter cloacae* (25.53%). The following species were only present in the absence of *P. aeruginosa*: *Enterobacter sakazakii*, *Klebsiella pneumoniae*, *Klebsiella oxytoca*, *Morganella morganii*, *Neisseria meningitidis*, *Proteus vulgaris*, *Serratia marcescens*, *Serratia liquefaciens*, *Bacillus cereus*, *Streptococcus oralis*, Viridans Streptococci, group F *Streptococcus* and *Aspergillus fumigatus.* The following species were only cultured in patients with *P. aeruginosa*-positive burn infections: *Acinetobacter lwoffii*, *Escherichia vulneris*, *Leclercia adecarboxylata*, *Proteus mirabilis*, *Pseudomonas putida*, *Raultella planticola*, *Streptococcus milleri*, *Candida dubliniensis* and *Candida glabrata*.

### 3.3. Environment

**Figure 1:**
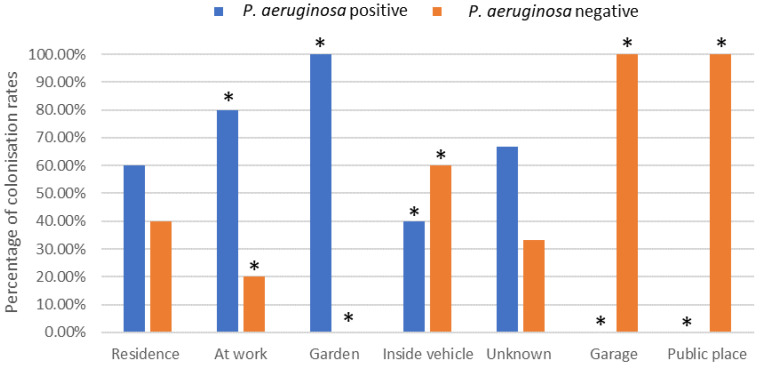
Effect of environmental location of burn incident on *P. aeruginosa* colonisation rates (%). * Indicates significant difference of *p* < 0.05.

The environment at injury was categorised into seven groups. There was a significant difference in the number of *P. aeruginosa*-positive and *P. aeruginosa*-negative patients that acquired their burn at work (80% positive, 20% negative), in a garden (100% positive, 0% negative), inside a vehicle (40% positive, 60% negative), in a garage (0% positive, 100% negative) or in a public place (0% positive, 100% negative) (*p* < 0.05) (Figure 1).

### 3.4. Clothing Fire

**Figure 2:**
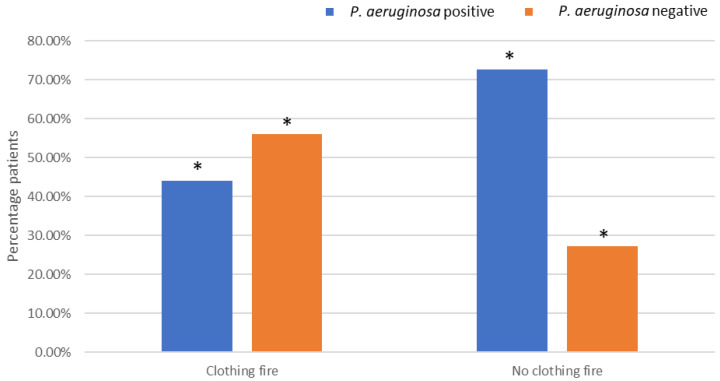
Effects of being involved in a clothing fire on *P. aeruginosa* colonisation. * Indicates significant difference.

*P. aeruginosa* isolation was significantly less common in clothing fire patients, with 56.0% of *P. aeruginosa*-negative patients having a documented clothing fire compared to 44.0% of *P. aeruginosa*-positive patients (Χ^2^(1) = 3.95, *p* = 0.05, OR = 0.30) (Figure 2). The opposite trend was demonstrated in patients that had not been involved with a clothing fire, whereby significantly more patients (72.7%) resulted in the isolation of *P. aeruginosa* bacteria from the wound compared to 27.0% that did not result in the isolation of *P. aeruginosa*.

### 3.5. Number of Surgeries

**Figure 3:**
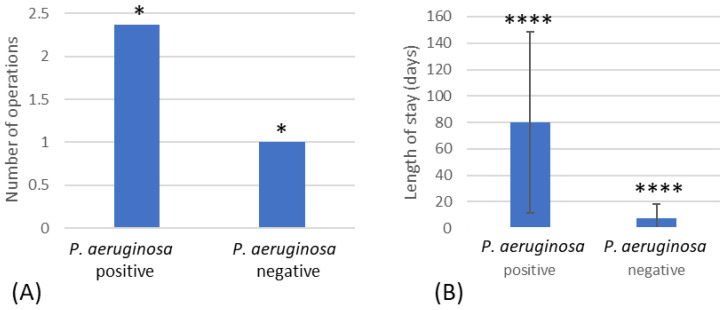
(**A**) Number of operations that resulted in *P. aeruginosa*-positive and -negative patients. (**B**) The length of stay for patients positive for *P. aeruginosa* compared to patients negative for *P. aeruginosa*. * Indicates significant difference of *p* < 0.05 and **** significant difference of *p* < 0.001.

The number of operations was higher in the patients that were positive for *P. aeruginosa* (2.4) compared to the *P. aeruginosa*-negative group (1.0) (Figure 3A). The length of stay, measured in days, was significantly increased in the patients positive for *P. aeruginosa* (80.1) compared to the *P. aeruginosa*-negative group (7.6) (*p* =< 0.001) (Figure 3B).

### 3.6. Mental Health Illness and Substance Abuse

**Figure 4:**
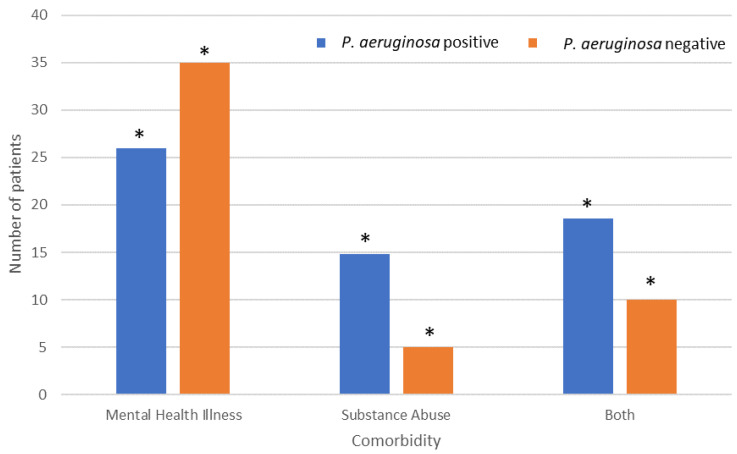
Effect of comorbidities on *P. aeruginosa* colonisation rates amongst patients with isolated *P. aeruginosa*. * Indicates significant difference.

Mental health illness and substance abuse were the most common comorbidities observed (Figure 4). Mental health illness demonstrated significantly fewer *P. aeruginosa*-positive patients (25.9%) than *P. aeruginosa*-negative patients (35%). However, patients that suffered from substance abuse demonstrated significantly higher numbers of isolated *P. aeruginosa* (14.8%) than non-isolated *P. aeruginosa* (5.0%). Patients that suffered from both mental health illness and substance abuse also demonstrated significantly higher numbers of *P. aeruginosa* (18.5%) than wounds where *P. aeruginosa* was not isolated (10.0%).

### 3.7. Mortality

**Figure 5:**
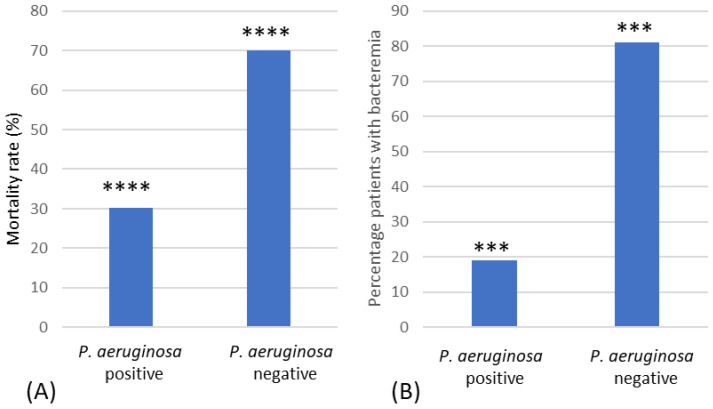
Mortality rate of burn patients. (**A**) Mortality rates (%) of *P. aeruginosa*-positive and -negative patients. (**B**) Mortality rates (%) of patients with *P. aeruginosa* bacteraemia and those who were *P. aeruginosa*-negative. *** Indicates significant difference of *p* < 0.005, **** significant difference of *p* < 0.001.

The mortality rate was significantly higher in the *P. aeruginosa*-negative group compared to the *P. aeruginosa*-positive group (Χ^2^(1) = 10.82, *p* = 0.001, Φ = +0.48) (Figure 5A), with an odds ratio of 9.63 (95% CI = 2.25–41.27). This remained significant when patients that died or were discharged within 7 days were removed (*p* = 0.008, Fisher’s exact test). *P. aeruginosa* bacteraemia was found in 19.0% of positive patients (Figure 5B), and these patients had a significantly lower mortality rate than *P. aeruginosa*-negative patients (*p* = 0.002, Fisher’s exact test) (Figure 5B).

## 4. Discussion

Although there have been many improvements in the care of burn patients, sepsis is the major cause of multiorgan failure triggers due to a dysregulated host response to infection [10]. However, the factors that influence the incidence of infection in burn patients is still unclear. The present study found a higher incidence of *P. aeruginosa* recovered from burn-wound infections. This coincides with other studies which have demonstrated that coagulase-negative Staphylococci spp. and *S. aureus* are the most prevalent isolates on admission wound cultures, with a shift in later weeks to *P. aeruginosa* as the most common isolate [11]. Gram-positive bacteria are able to survive the thermal insult and quickly colonise the burn wound within the first 48 h; these wounds are then subsequently colonised (5–7 days) with other opportunistic pathogens, including Gram-positive, Gram-negative and fungi. The source of these opportunistic pathogens includes the hosts microbiome or the hospital environment (i.e., transmission via a healthcare professional’s hands) [12].

In the current retrospective study, it was found that there were 39 different species of bacteria and five different species of fungi cultured, with 23 species of bacteria being Gram-negative and 16 being Gram-positive. The most commonly isolated organisms were *S. aureus*, *E. faecalis*, *E. coli*, *C. albicans*, *E. cloacae*, *S. maltophilia*, MRSA, *A. baumanii* and *C. freundii.* In studies by others, when burn wounds were swabbed at different skin depths, it was demonstrated that the most frequent cause of infection was found to be *S. aureus*, followed by *P. aeruginosa*, *K. pneumonia*, *E. coli* and *Acinetobacter* species [13]. In work on the bacterial species recovered from children in Tunisia, the most commonly isolated microorganisms were methicillin-susceptible *S. aureus*, *P. aeruginosa* and *E. cloacae* [14]. It was previously reported that the types of bacteria that colonise and infect burn patients were highly variable between burn units [15]; this could, in part, explain some of the differences observed between the results.

Initially, endogenous Gram-positive skin commensals may colonise the burn wound since they occur in the depths of sweat glands and hair follicles [16,17]. This may be followed by colonisation of microbial species from the patients’ gastrointestinal microbiome, such as *E. coli*, *Enterobacter* spp. and *Enterococcus* spp. [18]. The above species may also be transmitted from exogenous sources such as contaminated hands of healthcare workers, fomites, water or air [19].

In work by others, a total of 127 bacterial pathogens were isolated from 100 patients. Of these, most were monomicrobial, but a number were polymicrobial [13]. In this retrospective study, several other bacterial species were commonly isolated from burn patients who were often *P. aeruginosa*-positive, including *S. aureus*, *E. faecalis*, *E. coli*, *C. albicans* and *E. cloacae*. In comparison to single-species infections, bacteria may interact with each other, forming multispecies biofilms that are extremely difficult to eradicate due to the difficulty in antimicrobial penetration and the occurrence of persister cells. For example, the transmission of aminoglycoside resistance from one *Pseudomonas* spp. to another and to other Gram-negative organisms including *Enterobacter* spp., *Acinetobacter* spp., *E. coli* and others has been recognized in the burn population [15]. In addition, the importance of the coculture environment has been shown to influence bacterial virulence, but this subject is still controversial. For example, in the presence of *A. fumigatus*, the production of *P. aeruginosa* elastase was enhanced [20]; however, *A. fumigatus* supernatants have also been shown to demonstrate strong antipseudomonal activity [21], thus suggesting that understanding the complex microbiota of microorganisms found in burn wounds requires further investigation.

*Candida albicans* and to a lesser extent *Candida parapsilosis* were recovered from the wounds of the burns patients. Work by others showed that most fungal infections were in patients with massive burn injuries and were caused by *Candida* spp. [15]. In other work, the predominant yeast recovered from burn patients was found to be *C. krusei* [22], whilst in work by Capoor et al. [23], both *C. parapsilosis* and *A. niger* were recovered. *Aspergillus fumigatus* was recovered from five patients. It was demonstrated that burn patients with atypical invasive fungal infections can have severe concomitant polymicrobial infections and multidrug resistance with fatal results [24]. The past decade has demonstrated increased burn wound infections with atypical invasive fungal organisms, and non-*Candida* genera have included *Aspergillus* spp. [24].

There was a significant difference in the number of *P. aeruginosa* found in patients that acquired their burn at work, in a garden, inside a vehicle, in a garage or in a public place. However, due to a lack of more detailed information about the place where the burn was acquired, it is difficult to determine why this was the case. There was also significantly less *P. aeruginosa* isolated from patients that had been involved in clothing fires. Factors such as the clothing material and how tightly it fits the body may influence the protectiveness of the clothing [25], and it has been suggested that the clothing type may affect the extent of the burn in the victim. For example, in an observational study on clothing characteristics, it was demonstrated that wearing long, loose, flowing garments resulted in clothing catching fire more easily, and this resulted in more sustained burn injuries when compared to clothes reaching down to the knee and if the item of clothing was more in the style of a short-fitting dress [25]. In addition, the percentage of burns was found to be higher among wearers of synthetic fabrics when compared to fabrics that were made of cotton [25]. However, the relationship between clothing and the incidence of *P. aeruginosa* isolation from burn wounds still remains unclear.

Acute mental disorders in burn patients are associated with poor clinical outcomes, and preburn drug abuse and alcoholism was shown to increase the risk of acute mental disorders during initial treatment [26]. In addition, many burn survivors suffer from psychiatric conditions long after their physical injuries have healed, and this may be more pronounced in individuals who have a history of mental health disorders prior to admission [26]. This is of importance since the development of acute mental disorders in burn patients was associated with poor clinical outcomes including decreased survival rates, longer hospitalization times and increased complication rates [27]. This retrospective work demonstrated that patients that suffered from substance abuse and patients that suffered from combined mental health illness and substance abuse demonstrated significantly higher numbers of isolated *P. aeruginosa* from the wounds. During this retrospective study, petrol was used directly on the skin as part of a self-injury process in eight patients. However, Mahendraraj et al. [26] demonstrated that only a small percentage of burn patients were diagnosed with acute mental disorders at the time of the burn. Hence, in future studies, it might be prudent to follow up on the mental health of patients following treatment. A study by Nam et al. [27] demonstrated that patients with mental health disorders were younger, had larger burns and had significantly longer lengths of stay which resulted in significantly higher costs; however, it was also found that patients with preexisting mental health disorders had decreased odds of mortality [27]. In contrast, Mahendraraj et al. [26] demonstrated that acute mental disorder patients had a significantly longer length of hospitalization and shorter actuarial survival. The difference in these results may be due to the average age of the patient that was treated.

In this study, the mortality rates were found to be significantly lower in *P. aeruginosa*-positive patients compared to *P. aeruginosa*-negative patients and remained so when looking only at *P. aeruginosa* bacteraemia. However, due to the low numbers of major burns in services, firm conclusions can only be made using national-level data. The association that *P. aeruginosa* can decrease mortality is counterintuitive; this result may simply be due to the fact that *P. aeruginosa* is a later coloniser of burn wounds, and hence, patients that may contract such infections have survived for a longer period of time. It may also be that because a longer infection time leads to greater exposure of the patents to antibiotics, there is a relationship with *P. aeruginosa* colonisation, but this requires further investigation.

The Incidence of *P. aeruginosa* isolated from burn infections was associated with length of hospital stay, and this was also demonstrated previously by Wanis et al. [10]. However, their work found that Enterobacteriaceae isolation was highest between 7 and 14 days of hospitalization and that antibiotic resistance was directly proportional to hospital length of stay, with multidrug-resistant Gram-negative bacteria increasing from 6% at 7 days to 44% at 28 days. In our study, in patients that were positive for *P. aeruginosa*, the number of operations was also found to be significantly higher, although our data suggest that this is likely to have been influenced by the patients’ length of stay (or vice versa).

## 5. Conclusions

This retrospective study found that there were a number of significant factors that were related to the incidence of *P. aeruginosa* isolation from burn wounds. In agreement with others, there was a significant incidence of *P. aeruginosa* isolated with an increased length of hospital stay and patients having acute mental health disorders. In addition, the environment in which the patients acquired their burns, e.g., at work, in a garden, inside a vehicle, in a garage or in a public place, significantly influenced the incidence of isolated *P. aeruginosa*. Burns resulting from clothing fires significantly decreased the incidence of *P. aeruginosa* isolated from the burn wounds. Hence, further work is warranted on how the environment in which a burn is acquired influences the microbial consortia of the wound. Further, elucidation of the colonisation process, especially in the case of polymicrobial burn wounds, could result in the development of more effective treatment strategies.

## Figures and Tables

**Table 1:** Factors tested to determine a relationship with patients who tested positive for *P. aeruginosa*.

Factor	Subgroup	Significance
Species of microorganisms	Gram-negative bacteria	N/A
	Gram-positive bacteria	N/A
	Yeast and fungi	N/A
Environment	Inside a place of residence	No
	Inside a vehicle	Yes
	In a public place	Yes
	At work	Yes
	In a garden	Yes
	In a garage	Yes
	Unknown location	No
Location of burn	Scalp	No
	Face and head	No
	Neck	No
	Chest	No
	Abdomen	No
	Upper limb	No
	Hands	No
	Back	No
	Buttocks	No
	Perineum and genitalia	No
	Lower limb	No
	Feet	No
Type of burn	Flame burns	Numbers too small
	Scalds	Numbers too small
	Flash burns	Numbers too small
	Contact burns	Numbers too small
	Radiation burns	Numbers too small
	Explosions	Numbers too small
	Clothing fire	Yes
Hospitals	Admission from other hospitals	Numbers too small
Intubation	The number of intubated patients	No
	Location of intubation	No
Surgeries and length of stay	Number of surgeries	Yes
	Length of stay	Yes
First aid	Unknown	Numbers too small
	Water	Numbers too small
	Cling film	Numbers too small
Co-morbidities	Mental health issues	Yes
	Substance abuse	Yes
	Mental health issues and substance abuse	Yes
Mortality	Mortality rate	Yes
	Mortality with *P. aeruginosa* bacteraemia	Yes
	Age	No
	Total body surface area (%)	No
Type of antibiotic	Tazocin	No
	Meropenem	No
	Vancomycin	No
	Clindamycin	No
	Amphotericin	No
	Colomycin	No
	Clarithromycin	No
	Co-trimoxazole	No
	Gentamicin	No
	Flucloxacillin	No
	Benzylpenicillin	No
	Co-amoxiclav	No
	Cefuroxime	No
	Erythromycin	No
	Tobramycin	No
	Amoxicillin	No
	Ciprofloxacin	No

**Table 2:** Bacterial and fungal species isolated from burn wounds of patients.

	*P. aeruginosa*-Positive Culture (%)	*P. aeruginosa*-Negative Culture (%)		*P. aeruginosa*-Positive Culture (%)	*P. aeruginosa*-Negative Culture (%)
*Acinetobacter baumannii*	14.8	5	*Bacillus cereus*	0	5
*Acinetobacter lwoffii*	3.7	0	*Clostridium difficile*	3.7	0
*Aeromonas hydrophila*	3.7	5	*Enterococcus faecalis*	40.7	30
*Citrobacter freundii*	14.8	5	*Enterococcus faecium*	11.1	10
*Cronobacter sakazakii*	0	5	*Staphylococcus aureus*	40.7	55
*Enterobacter cloacae*	29.6	20	*Staphylococcus epidermidis*	3.7	5
*Escherichia coli*	33.3	30	Methicillin-resistant *Staphylococcus aureus* (MRSA)	18.5	10
*Escherichia vulneris*	3.7	0	*Streptococcus milleri*	3.7	0
*Haemophilus influenzae*	14.8	5	*Streptococcus pneumoniae*	11.1	10
*Klebsiella pneumoniae*	0	15	*Streptococcus oralis*	0	5
*Klebsiella oxytoca*	0	10	Viridans group streptococci (VGS)	0	5
*Leclercia adecarboxylata*	7.4	0	Group A Streptococcus	0	5
*Moraxella catarrhalis*	3.7	5	Group B Streptococcus	11.1	10
*Morganella morganii*	0	5	Group C Streptococcus	3.7	10
*Neisseria meningitidis*	0	5	Group G Streptococcus	3.7	5
*Proteus mirabilis*	3.7	0	Group F Streptococcus	0	5
*Proteus vulgaris*	0	5			
*Pseudomonas aeruginosa*	57.45	43	Fungi and yeast		
*Pseudomonas putida*	3.7	0	*Aspergillus fumigatus*	0	5
*Raoultella planticola*	3.7	0	*Candida albicans*	29.7	25
*Serratia marcescens*	0	5	*Candida dubliniensis*	3.7	0
*Serratia liquefaciens*	0	5	*Candida glabrata*	3.7	0
*Stenotrophomonas maltophilia*	18.5	10	*Candida parapsilosis*	11.1	5

## Data Availability

The data presented in this study are available on request from the corresponding author.

## References

[1] Whitaker I.S., Shokrollahi K., Dickson W.A. (2019). Burns (Oxford Specialist Handbooks in Surgery).

[2] Sevgi M., Toklu A., Vecchio D., Hamblin M.R. (2013). Topical antimicrobials for burn infections—An update. Recent Pat. Anti-Infect. Drug Discov..

[3] Krishnan P., Frew Q., Green A., Martin R., Dziewulski P. (2013). Cause of death and correlation with autopsy findings in burns patients. Burns.

[4] Azzopardi E., Azzopardi E., Camilleri L., Villapalos J., Boyce D.E., Dziewulski P., Dickson W.A., Whitaker I.S. (2014). Gram negative wound infection in hospitalised adult burn patients—Systematic review and metanalysis. PLoS ONE.

[5] Strateva T., Yordanov D. (2009). *Pseudomonas aeruginosa*—A phenomenon of bacterial resistance. J. Med. Microbiol..

[6] Yoon S.S., Hennigan R.F., Hilliard G.M., Ochsner U.A., Parvatiyar K., Kamani M.C., Allen H.L., DeKievit T.R., Gardner P.R., Schwab U. (2002). *Pseudomonas aeruginosa* anaerobic respiration in biofilms: Relationships to cystic fibrosis pathogenesis. Dev. Cell.

[7] Mah T.-F., Pitts B., Pellock B., Walker G.C., Stewart P.S., O’Toole G.A. (2003). A genetic basis for *Pseudomonas aeruginosa* biofilm antibiotic resistance. Nature.

[8] DeLeon S., Clinton A., Fowler H., Everett J., Horswill A.R., Rumbaugh K.P. (2014). Synergistic interactions of Pseudomonas aeruginosa and Staphylococcus aureus in an in vitro wound model. Infect. Immun..

[9] Maslova E., Eisaiankhongi L., Sjöberg F., McCarthy R. (2021). Burns and biofilms: Priority pathogens and in vivo models. NPJ Biofilms Microbiomes.

[10] Wanis M., Walker S.A.N., Daneman N., Elligsen M., Palmay L., Simor A., Cartotto R. (2016). Impact of hospital length of stay on the distribution of Gram negative bacteria and likelihood of isolating a resistant organism in a Canadian burn center. Burns.

[11] Altoparlak U., Erol S., Akcan M.N., Celebi F., Kadanali A. (2004). The time-related changes of antimicrobial resistance patterns and predominant bacterial profiles of burn wounds and body flora of burned patients. Burns.

[12] Church N.A., McKillip J.L. (2021). Antibiotic resistance crisis: Challenges and imperatives. Biologia.

[13] Shareen George K.G., Basavarajappa A.R., Hanumanthappa A.R. (2015). Bacteriological study of burns infection. J. Evol. Med. Dent. Sci..

[14] Hassen A.F., Khalifa S.B., Daiki M. (2014). Epidemiological and bacteriological profiles in children with burns. Burns.

[15] Tredget E.E., Shankowsky H.A., Joffe A.M., Inkson T.I., Volpel K., Paranchych W., Kibsey P.C., Alton J.D.M., Burke J.F. (1992). Epidemiology of infections with *Pseudomonas aeruginosa* in burn patients: The role of hydrotherapy. Clin. Inf. Dis..

[16] Barret J., Herndon D. (2003). Effects of burn wound excision on bacterial colonization and invasion. Plast. Reconstr. Surg..

[17] Bang R.L., Gang R.K., Sanyal S.C., Mokaddas E.M., Lari A.R.A. (1999). Beta- haemolytic Streptococcus infection in burns. Burns.

[18] Ramzy P., Wolf S., Irtun O., Hart D.W., Thompson J.C., Herdon D.N. (2000). Gut epithelial apoptosis after severe burn: Effects of gut hypoperfusion. J. Am. Coll. Surg..

[19] Weber J., Sheridan R., Pasternack M., Tompkins R.G. (1997). Nosocomial infections in pediatric patients with burns. Am. J. Inf. Con..

[20] Smith K., Rajendran R., Kerr S., Lappin D.F., Mackay W.G., Williams C., Ramage G. (2015). *Aspergillus fumigatus* enhances elastase production in *Pseudomonas aeruginosa* co-cultures. Med. Mycol..

[21] Reece E., Doyle S., Greally P., Renwick J., McClean S. (2018). *Aspergillus fumigatus* Inhibits *Pseudomonas aeruginosa* in Co-culture: Implications of a Mutually Antagonistic Relationship on Virulence and Inflammation in the CF Airway. Front. Microbiol..

[22] Mousa H.A.-L., Al-Bader S.M. (2001). Yeast infection of burns. Mycoses.

[23] Capoor M.R., Gupta S., Sarabahi S., Mishra A., Tiwari V.K., Aggarwal P. (2012). Epidemiological and clinico-mycological profile of fungal wound infection from largest burn centre in Asia. Mycoses.

[24] Akhavan A.A., Shamoun F., Lagziel T., Rostami S., Cox C.A., Cooney C.M., Sood G., Hultman C.S., Caffrey J.A. (2023). Invasive Non-*Candida* Fungal Infections in Acute Burns—A 13-Year Review of a Single Institution and Review of the Literature. J. Burn Care Res..

[25] Muguregowda H. (2019). An observational study on clothing characteristics involved as major contributors in sustaining domestic burns injuries. World J. Plas. Surg..

[26] Mahendraraj K., Durgan D.M., Chamberlain R.S. (2016). Acute mental disorders and short and long term morbidity in patients with third degree flame burn: A population-based outcome study of 96,451 patients from the Nationwide Inpatient Sample (NIS) database (2001–2011). Burns.

[27] Nam J., Sljivic S., Matthews R., Pak J., Agala C., Salamah H., Hatch E., Nizamani R., King B., Laughon S.L. (2023). The cost of mental health comorbid conditions in burn patients: A single-site experience. J. Burn Care Res..

